# The Latest Cellular and Molecular Mechanisms of COVID-19 on Non-Lung Organs

**DOI:** 10.3390/brainsci13030415

**Published:** 2023-02-27

**Authors:** Hamid Askari, Fatemeh Rabiei, Fatemeh Lohrasbi, Sara Ghadir, Maryam Ghasemi-Kasman

**Affiliations:** 1 Student Research Committee, Babol University of Medical Sciences, Babol 47176-47745, Iran; 2 Cellular and Molecular Biology Research Center, Health Research Institute, Babol University of Medical Sciences, Babol 47176-47745, Iran; 3 Department of Physiology, School of Medicine, Babol University of Medical Sciences, Babol 47176-47745, Iran

**Keywords:** SARS-CoV-2, COVID-19, cellular mechanisms, molecular mechanisms, organ damage

## Abstract

Understanding the transmission pathways of severe acute respiratory syndrome coronavirus 2 (SARS-CoV-2) will aid in developing effective therapies directed at the virus’s life cycle or its side effects. While severe respiratory distress is the most common symptom of a coronavirus 2019 (COVID-19) infection, the virus is also known to cause damage to almost every major organ and system in the body. However, it is not obvious whether pathological changes in extra-respiratory organs are caused by direct infection, indirect, or combination of these effects. In this narrative review, we first elaborate on the characteristics of SARS-CoV-2, followed by the mechanisms of this virus on various organs such as brain, eye, and olfactory nerve and different systems such as the endocrine and gastrointestinal systems.

## 1. Introduction

The first case of SARS-CoV-2 was officially reported in Wuhan, China, in 2019 [1]. SARS-CoV-2 is a member of the family Coronaviridae, and its RNA is encapsulated and enveloped [2]. Fusion and attachment of the virus to the cell surface are facilitated by the spike (S) glycoprotein [3]. The receptors for the virus to invade are Angiotensin-converting enzyme 2 (ACE-2) receptors. Furthermore, the host cell’s transmembrane serine protease 2 (TMPRSS2) is essential for priming the viral S protein [4].

Besides lung infection, most of the vital organs can be infected by SARS-CoV-2, for instance, the brain, olfactory nerve, eye, and endocrine and gastrointestinal systems [5]. In addition to respiratory distress, the most prominent symptom of COVID-19 that patients often experience are nausea, vertigo, and headache, which implies a possible involvement of the neurological system [6]. Also, post-COVID-19 symptoms include fatigue and cognitive impairment [7,8]. Evidence from the acute infection and the long-term effects of COVID-19 show a role for the senses of smell and taste. Mainly, they include a complete or partial lack of smell (anosmia/hyposmia) and taste (ageusia/hypogeusia) or a distorted perception of smell/taste (parosmia/parageusia) or the sense of an odor or a taste without any concomitant stimulus (phantosmia, also known as olfactory hallucination, and phantogeusia, also known as a gustatory hallucination) [8,9]. Furthermore, patients experience digestive symptoms such as vomiting, diarrhea, nausea, and abdominal discomfort [10]. In addition, recent developments suggest that COVID-19 individuals’ endocrine systems may be affected [11]. 

Identifying, treating, and managing COVID-19 cases effectively require knowledge of the most up-to-date cellular and molecular processes. These mechanisms identified in this literature review suggest possible links between SARS-CoV-2 infection and organs dysfunction.

## 2. SARS-CoV-2 Characteristics

**Figure 1:**
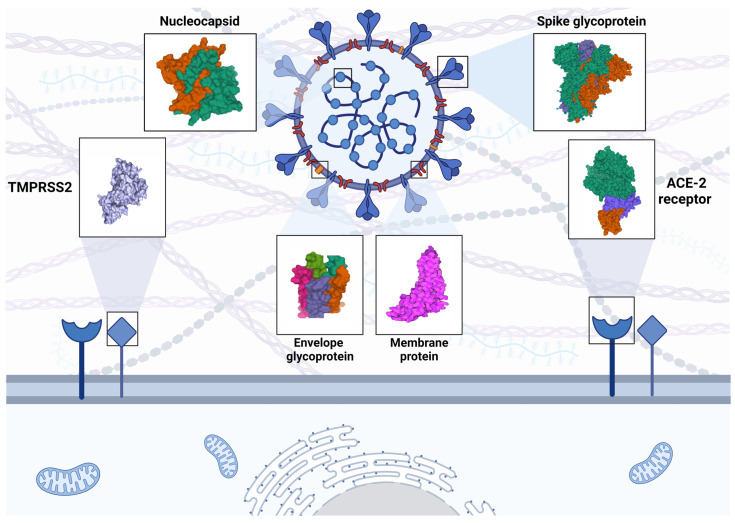
SARS-CoV-2 characteristics. The proteins S glycoprotein, E glycoprotein, M, and N are required to SARS-CoV-2 pathogenicity, replication, and structure. The virus uses ACE-2 receptors to enter cells. Priming the viral S protein requires the host cell’s TMPRSS2. (Created with BioRender.com).

The SARS-CoV-2 is a single-stranded RNA virus that is encapsulated [2]. It belongs to the genus Betacoronavirus, the family Coronaviridae, the subfamily Coronavirinae, and the order Nidovirales [12]. The genome size of the virus, which is 29.99 kB, is significant [13]. As with other coronavirus genomes, the SARS-CoV-2 genome contains open reading frames (ORF). Approximately 67% of the genome is encoded by ORF1ab. This coding may allow infected cells to create polyproteins. Both nsp3 and nsp5 are proteases that break down polyproteins into 16 different nonstructural proteins: nsp1–11 from the ORF1a segment and nsp12–16 from the ORFb segment [14]. The other 33% of the genome is comprised of supplementary and structural (additional) protein genes (ORF3a, 3b, 6, 7a, 7b, 8, 9b, and 10) [13,14]. The virulence, replication, and structure of SARS-CoV-2 are all dependent on the proteins S glycoprotein, small envelope (E) glycoprotein, membrane (M), and nucleocapsid (N) (Figure 1) [15,16]. After being delivered to the host cell membrane via vesicles, viral assemblies are exocytosed. S-proteins enable the fusion of infected and healthy cells when delivered to the cell surface. Enormous, multinucleated cells are produced, spreading the virus throughout the host [17]. 

Multiple resuscitation syndromes are characterized by systemic inflammation. They are associated with viral replication, cellular breakdown products entering the circulation, hypoxia, pathologically enhanced macrophage and T-cell activity, complement systems, leukocyte activation inside the vasculature, and hemostasis [18]. 

Viruses are distinguished by the fact that they suppress the receptor signaling pathways that initiate antiviral immunity. These conserved molecular structures are pattern recognition receptors (PRRs), which identify pathogen-associated molecular patterns and damage-associated molecular patterns (PAMP and DAMP). DAMPs are produced by host cells that are injured or dying, while PAMPs originate from pathogens. Toll-like receptors (TLR) are the most prominent family of PRRs that detect endosomal viral RNA, whereas RIG-I-like receptors (RLR) detect cytoplasmic viral RNA [19]. When these receptors are engaged, protection against viruses triggered by innate immunity, predominantly related to the generation of interferons (IFNs), is induced. Scavenger receptors (SR) are a unique family of molecules that may interact with viruses in a non-specific way [20]. This vast family of receptors is at the crossroads of metabolism and immunology. Typically, they are located on dendritic cells and stromal macrophages [21]. SRs can be cofactors of TLRs in innate immune cells’ identification and neutralization of viruses. Nevertheless, they may also act as an entrance site for virus [22]. 

Coronavirus S-glycoproteins, which facilitate membrane fusion and viral penetration, bind to host receptors [23]. Membrane ACE-2 is the most important receptor for viruses, of which one of the two ACE-2 isoforms is unable to bind [24]. On the surface of the virus, S-glycoprotein forms trimers. Following receptor-binding domain (RBD) receptor contact, the S-protein is proteolytically cleaved by the host proteases into the N-terminal S1 subunit and the C-terminal S2 subunit. TMPRSS2 SRs are the catalyst for this partial proteolysis, which can be initiated by furin or furin-like proteases (like plasmin) or by cathepsins B/L following endocytosis (Figure 1) [25]. Direct interactions between the S1 subunit and ACE-2 occur at its RBD. It is possible that one of the three RBDs in the S-protein trimer may be twisted into a conformation that permits its connection to the receptor. The S-glycoprotein carbohydrate component protects the RBD against antibodies [26]. Thus, SARS-CoV-2 remains in a state of low binding activity with ACE-2 for a considerable time. Consequently, receptor-binding determinants undergo a conformational hinge movement that momentarily conceals or reveals them [27]. Antibodies and medications that target RBD binding cannot completely eradicate SARS-CoV-2 due to this property.

Thus, upon receptor binding, the virus undergoes proteolytic processing that activates the S-protein, bringing the viral and host membranes together and releasing RNA of virus into the host cell cytoplasm. The anchoring S2 subunit facilitates virus-host cell membrane fusion, whereas the distal S1 subunit recognizes and binds the receptor [28]. SARS-CoV-2 is very likely to take this route [29]. The endosomal pathway can be activated by many co-receptors collaborating with ACE-2 [26]. 

Recently, several membrane proteins capable of functioning as alternative receptors or ACE-2 cofactors have been identified. Therefore, the oligomannose and complex sugars in the carbohydrate component of the S-glycoprotein, which is made of N-glycans of the S1 subunit, may bind to lectin receptors and interact with them to shield the virus from antibodies [30,31]. Heparin binds to S1 sites that resemble lasting, perhaps preventing or slowing the viral invasion [32]. The glycocalyx of the infected cell provides a binding site for the virus due to the expression of co-receptor sugars for binding the virus (heparan sulfate and O-acetylated sialic acids) [33]. Invasion may be aided by lectin-like S1 sites attaching to the glycocalyx of the host cell. Evidence suggests that heparan sulfate facilitates the entry of many viral types [34]. The lectin-like S1 domain of the ACE-2 receptor may interact with the glycocalyx during infection of target cells, which may alter the receptor’s function [33,35]. The S protein binds to the RGD motif of the RBD S1 domain of integrin receptors (Arg-Gly-Asp). The role of these interactions in viral neutralization or invasion sometimes unclear [36].

Several RNA viruses use extracellular vesicles for transmission to new hosts [37]. Viruses can infect cells independently and through virus-specific receptors due to these vesicles. After treatment with antiviral drugs, infection recurrence has been linked to SARS-CoV-2 and the cellular transport pathway involved in generating extracellular vesicles carrying the virus [38]. COVID-19 transmission may include exosomes containing ACE-2, the cluster of differentiation (CD9), and other tetraspanins [39]. Because exosomes can transport particles of virus from infected to normal tissues and regulate the host immune system, they can also be employed to treat COVID-19 [40].

There was insufficient evidence to support the hypothesis that CD147 served as an ACE-2-independent SARS-CoV-2 receptor in the first investigations of this function. The CD147 mechanism is indirect and linked to membrane ACE-2 expression [41]. Simultaneously, it has been demonstrated that the scavenger receptor SR-B1, which identifies high-density lipoprotein (HDL), facilitates SARS-CoV-2 entry in an ACE2-dependent manner. The viral S1 subunit increases viral uptake by binding to HDL and cholesterol components. When SR-B1 was expressed, SARS-CoV-2 could enter cells expressing ACE-2 with greater ease due to the interaction between SR-B1 and these receptors. In human lung tissue and various extrapulmonary tissues, it has been reported that SR-B1 and ACE-2 are co-expressed [22]. SR-B1 is involved in COVID-19 due to its immunomodulatory, virus penetration intermediary, and viral scavenger activities [42].

C-type lectin CD209L (L-SIGN) could function as its own SARS-CoV-1 receptor on target cells [43]. Researchers were intrigued by the finding that CD209L and the similar protein CD209 (DC-SIGN) function as receptors that allow SARS-CoV-2 entering to the human cells [44]. 

People with severe COVID-19 infections had significantly elevated complement activation in their lung tissue, skin, and serum [45]. Multiple mechanisms contribute to the activation of COVID-19’s complement system. Most of these processes include vascular endothelial cell injury, activation of hemostasis systems, and kallikrein-kinins [46]. 

S-protein binds to TLR4 and activates it, which enhances ACE-2 on the cell surface, allowing the virus to enter type II alveolocytes. This disrupts the production of surfactant and destroys cells, all contributing to the onset of ARDS. The SARS-CoV-2 that causes myocarditis is linked to TLR4 activity and the resultant upregulation of the innate immune reaction [47]. Molecular docking experiments show that S-protein binds to TLR1 and TLR6 but not TLR4 [48]. 

Temporarily formed during single-stranded viral RNA production, double-stranded viral RNA may attach to macrophages and dendritic cells via TLR3, but TLR7 and TLR8 can bind to single-stranded RNA [49]. Endosomal TLRs produce IFN-I responses and cytokine production, suppressing viral infection [50]. 

The RLR/MAVS-mediated signaling system is important for the antiviral response in individuals with COVID-19, and its dysfunction may cause autoimmune disorders and a cytokine storm [51]. Furthermore, many cells undergo viral-vague cell stress pathways, including NF-B activation and NOD like-receptor protein (NLRP3) inflammasome association, resulting from SARS-CoV-2’s action on the NLR and potential modifications in intracellular homeostasis [52]. On the other hand, inflammasomes boost interleukin (IL)-1 and IL-18 production and cause the cell to undergo pyroptosis [53]. The inflammasome may have either protective or pathogenic effects, depending on the context [54]. 

SARS-CoV-2 target cells are found by cell antiviral defense sensors, and vice versa, at the earliest stage of the virus’s invasion. Since, the ACE-2 and coreceptors, as well as its other receptors, become active. This wide range of hosts means that viruses can infect cells in many different parts of the body. When viruses enter a cell, they turn on some stress signaling pathways, some of which are universal and some specific to the virus. Additionally, there is a rise in the reciprocal antagonism between immune systems, most of which are IFN-dependent, and the preexisting system of the virus’s critical components, both at the cellular and organismal levels (the virus and recruited proteins). The result of the conflict will determine how far the illness spreads.

There are several types of coronaviruses, and it is important to note that they vary from strain to strain. The SARS-CoV-2 main variants, B.1.1.7 (alpha), B.1.351 (beta), P.1 (gamma), and B.1.617.2 (delta), have already spread to a wide range of populations around the world. However, certain novel variants are distinguished by newly mutated nucleotides with unknown influence on infectivity, mortality, and antigenicity [55]. Nevertheless, this study focuses primarily on COVID-19 and the chemical pathways that lead to its induction.

## 3. Investigating Neurological Disorders

### 3.1. Potential SARS-CoV-2-Mediated Brain Injury Mechanisms

The likely origins of neurodegeneration are illuminated by the pathophysiology of SARS-CoV-2 and other human coronaviruses (HCoV). The SARS-CoV-2 invasion hypothesis involves a cell protease-primed viral S protein and a cell surf-receptor. SARS-CoV-2 particularly employs TMPRSS2 cell protease for S protein priming and ACE-2 as its entrance receptor [56]. Oligodendrocytes, ciliated epithelial cells, and nasal goblet cells were shown to co-express ACE-2 and TMPRSS2 in cross-sectional analyses of human tissues [56]. One strategy for central nervous system (CNS) infiltration or proliferation in oligodendrocytes could be the co-expression of ACE-2/TMPRSS2. During the pandemic, cases of acute encephalitis were reported, and patients’ cerebrospinal fluid (CSF) contained the virus [57]. Pathological studies, in which both infectious viruses and viral RNA have been found in the brain, provide additional insight. Antigen and RNA of the virus were found in the cerebrums of the SARS-CoV-infected individuals in a postmortem examination of four SARS-CoV-related deaths and four controls [58]. Previous autopsy investigations have detected the following other human coronaviruses in the brain: 44 of 90 brain donors had the HCoV strains 229E and OC43, as identified by reverse transcription-polymerase chain reaction (RT-PCR) [59]. Notably, the prevalence of OC43 was significantly higher in multiple sclerosis (MS) patients compared to controls. Monocyte chemoattractant protein (MCP)-1 chemokine mRNA was also observed to be elevated in astrocyte cell lines after HCoV-OC43 infection [60]. The blood-brain barrier (BBB) has been shown to become more permeable when MCP-1 levels rise [61]. These results suggest that HCoV infection might enhance a tendency for MS neuropathology, and coronavirus infection can interact with underlying neuropathology to produce additive or permanent neurologic consequences.

The hematogenous and transneuronal pathways are viable entry points for coronaviruses into the CNS. Early onset of anosmia is a distinguishing feature of a virus that may be indicative of an early neuro-invasion via the olfactory bulb. On magnetic resonance imaging (MRI), atrophy of the olfactory bulb was associated with anosmia in a patient with COVID-19 [62]. Intriguingly, increased cytokine levels were detected in the olfactory epithelium of COVID-19 patients since this may indicate that direct inflammation of the olfactory tract may play a critical role in the onset of sensory loss [63]. Cases of encephalitis, neuronal ischemia, and SARS-CoV particles have all been related in clinical studies. During a human autopsy, researchers discovered genetic sequences in the brain [64]. Even though the genomes of SARS-CoV and SARS-CoV-2 are 82% identical to one another, SARS-CoV-2 contains distinct genetic traits such as the ability to encode proteins that can potentially influence viral replication and pathogenicity [65]. These genetic differences’ implications and significance are still unknown.

Coronaviruses may enter the CNS by several mechanisms, including inflammatory mediators, the transmigration of virus-carrying macrophages, direct infection of endothelial cells, and a BBB weakened by endothelial injury or endothelitis [59]. In transgenic mice models expressing human ACE-2 receptors, the SARS-CoV was found to rapidly transmit infection throughout the CNS and kill infected neurons after it had established itself in the nervous system [66]. Both infected and non-infected cells may perish by apoptosis after HCOV-OC43 infection of the hippocampus and cortical neural cells in tissue culture [67]. According to previous research, the infected cells released tumor necrosis factor (TNF-α), a known apoptotic cytokine, which may have contributed to apoptosis in uninfected cells as well as the infiltration and activation of microglia [68]. 

However, studies of evolutionary data imply that the new coronavirus may identify human ACE-2 more effectively [69]. SARS-CoV-2 and clinical-grade soluble human ACE-2 (hrsACE-2) were introduced into engineered human tissue in a study. The hrsACE-2 effectively scavenged the virus, blocking its cellular attachment [70]. 

Transgenic mice expressing human ACE-2 receptors were infected with SARS-CoV, and compared to wild-type mice, they developed more severe lung lesions, detectable viral antigens in the brain, inflammation in cerebral vessels, and hemorrhages. As a result, they were also more susceptible to contracting the virus [71]. 

In addition to acute lung damage, neurotoxicity may result from cytokine storms; Mice infected with the virus die within six days. Vascular hyperpermeability was seen in both the brain and lungs, and the cytokines IL-6, IL-1, and TNF- α were significantly elevated after infection with the virus [72]. Due to immune-mediated toxicity and cytokine-driven damage, the BBB’s integrity may be compromised even without viral invasion or dissemination [73]. Cytokines can also be directly neurotoxic, mediate of injury to CNS cells, or even prevent injury altogether [74]. It is unclear how the altered neuroinflammatory pathways following SARS-CoV-2 infection will highly activate the cytokine signaling that may affect neurologic outcomes.

### 3.2. COVID-19 Associated Neurological Symptoms

In contrast to recognized pulmonary symptoms, numerous neurological complications have been discovered following COVID-19 infection. Acute necrotizing encephalopathy, encephalitis, epilepsy/seizures, and ataxia are among the neurological manifestations are highly linked with COVID-19 acuity and mortality in intensive care unit patients [75]. Hypogeusia, hyposmia, Guillain-Barré syndrome (GBS), and skeletal muscle damage are all peripheral nervous system (PNS) consequences that have been reported [76]. 

#### 3.2.1. Cerebrovascular Diseases

Among the COVID-19 population, five percent had experienced cerebrovascular events, sixty percent of which were acute ischemic strokes [77]. The increased risk of these events has been associated with viral infection, which has been connected to a hyperinflammatory and hypercoagulable state and altered endothelial cell function [78]. The neutrophil-to-lymphocyte ratio (NLR), C-reactive protein (CRP), and serum ferritin have all been found to significantly rise in patients with ischemic stroke, suggesting that these patients’ mortality may be predicted [79]. These individuals have elevated neutrophils, leading to an overproduction of neutrophil extracellular traps (NETs), increasing thrombus development [80]. The SARS-CoV-2 mediated endothelial damage leads to nitric oxide synthase (NOS) consumption and a deficiency in nitric oxide (NO), promoting thrombus formation. The NO deficiency elevates the risk of stroke because it acts as a potent vasodilator and inhibits platelet and leukocyte adhesion to the endothelium [81]. 

In addition, as SARS-CoV-2 internalizes ACE-2 after binding, ACE-2 depletion on the surface of endothelial cells may enhance the risk of ischemic stroke [82]. SARS-CoV-2 patients have significantly lower levels of ACE-2 expression [83]. 

Since the current inpatient guidelines for managing and treating ischemic or hemorrhagic stroke are being reviewed, it is agreed that all COVID-19 patients in intensive care units (ICUs) should be assessed for thromboprophylaxis owing to the increased risk of stasis [84]. 

#### 3.2.2. Encephalitis

Meningitis and encephalitis both involve inflammation of the meninges and parenchyma of the brain [85]. The patient complains of a head pain, fever, vomiting, seizures, and reduced sensibility. Patients with COVID-19 who later experienced meningitis or encephalitis had SARS-CoV-2 in their CSF and brain tissues, suggesting that the virus itself may cause these complications by infecting and damaging the brain tissue [86]. 

Patients with COVID-19 may develop acute meningoencephalitis even if no detectable SARS-CoV-2 or other viruses are present in the CSF [87]. Meningoencephalitis in COVID-19 patients may be caused by other mechanisms, such as severe inflammation. In the treatment of COVID-19 patients, it ought to be taken into consideration as a potential complication due to the fatal effects of encephalitis and meningoencephalitis. Early diagnosis and treatment of meningoencephalitis are crucial for preventing deadly hemorrhagic encephalopathy.

After a patient has been diagnosed with encephalitis, meningoencephalitis, or acute neurological event (ANE), CSF PCR should be conducted to check for the presence of SARS-CoV-2 or other possible contributory viral diseases, such as herpes simplex virus (HSV). In addition, combining MRI and electroencephalography (EEG) is very important for the detection of these cases [88]. 

#### 3.2.3. Seizures

Some coronavirus patients will develop seizures due to hypoxia, metabolic disturbances, serious irritation, and organ disappointment. Seizures in patients have been associated with SARS-CoV-2-induced brain injury, elevated inflammatory mediator levels, and viral-induced encephalitis or meningitis [89]. In order to improve the diagnosis, treatment, and prevention of long-term problems with seizures in COVID-19 individuals, it is crucial to distinguish between the usual and atypical appearance of seizures [90]. 

#### 3.2.4. Guillain–Barré Syndrome (GBS)

The molecular resemblance between peripheral nerve antigens and pathogen antigens may lead to GBS after infections with Campylobacter jejuni, Epstein-Barr virus, or cytomegalovirus (CMV). Afterwards, neuronal injury and inflammation might occur due to antipathogen antibodies cross-reacting with peripheral nerve antigens [91]. Multiple cases of GBS in COVID-19 patients have been reported, characterized by paresthesia, weakness of the lower limbs, and the potential for tetraparesis [92]. Typically, nerve roots are involved, as evidenced by a normal white blood cell count and an elevated protein concentration in the CSF (cytoalbuminologic dissociation) [93]. Axonal injury and demyelinating polyradiculoneuropathy are classic features of GBS in COVID-19 patients [94]. 

#### 3.2.5. Neurodegenerative and Demyelinating Disorders

Multiple sclerosis (MS), Alzheimer’s disease (AD), and Parkinson’s disease (PD) are just a few of the neurodegenerative conditions that have been linked to SARS-CoV-2 infection. There is likewise no proof that these illnesses are accelerated in coronavirus patients [95]. However, the brain damage caused by the virus and the high expression of ACE-2 in the CNS may result in long-term neurodegenerative diseases or complications [96]. Demyelination of both gray and white matter as well as neurodegeneration in the brain’s periphery are related to the MS [97]. Based on what we know today, the neurological alterations brought on by SARS-CoV-2 have certain characteristics with MS. The first factor in neuroinflammatory injury is the pro-inflammatory ’cytokine storm’ brought on by SARS-CoV-2 infection [98]. The second issue is that SARS-CoV-2 might lead to demyelination in the CNS [99]. When immunotherapy is employed to treat demyelinating neurological illnesses like MS, the possibility of a link between SARS-CoV-2 infection and these diseases presents a difficult therapeutic conundrum [100]. However, no evidence exists to back up the allegations that viruses cause MS or that SARS-CoV-2-mediated immune dysregulation makes MS patients more vulnerable to COVID-19, its CNS involvement, or the return of MS symptoms [101,102]. In a 67-year-old MS patient who passed away from COVID-19, neither neuronal nor glial cells acquired the virus, and infection did not worsen or reactivate the patient’s symptoms [102]. The results of the preceding example are consistent with those of earlier research, which showed that COVID-19 had no impact on developing autoimmune illnesses [101]. 

PD patients, like those with AD, also have trouble remembering things [103]. Even though ACE-2 is extensively expressed in the CNS and SARS-CoV-2 infects and destroys various parts of the brain, there is no conclusive evidence that SARS-CoV-2 causes PD or that PD patients are more likely to receive SARS-CoV-2. There is also no evidence that PD worsens with COVID-19 disease [104]. 

Potential CNS damage that may lead to PD seems to be more restricted to the substantia nigra than in AD [105]. The pathophysiology of PD is similar to AD in that it involves neuroinflammation, synaptic pruning, and cell death [106,107]. Anosmia and hyposmia are also early clinical signs of PD [108]. Hence, further long-term studies are needed to discover the association between SARS-CoV-2 infection and PD, as well as for AD and other neurodegenerative disorders.

## 4. Complications of Skeletal Muscle and Neuromuscular Junction

COVID-19 patients’ reports show the involvement of PNS and its effect on neuromuscular deficiencies such as skeletal muscle injury and neuromuscular junction disorders. COVID-19 initiates impairment of the immune system, which contributes to PNS dysfunction [76]. Hypercatabolic situations associated with oxidative stress can lead to the extreme production of proinflammatory cytokines. Additionally, it can cause constructability enhancement of corrosive molecules, which gives rise to myocyte damage [109]. Myocytes exhibit the surface receptor ACE-2 that SARS-CoV-1 and SARS-CoV-2 use to enter the host cell. The connection between SARS-CoV-2 infection and the pathophysiologic processes that cause myopathy is not well understood, and additional study is required [110]. Demyelination caused by ischemia and inflammation after facial nerve paralysis may cause damage to the vasa nervorum [111].Studies illustrated that the invasion of the virus directly into the nerve could immune responses, nerve damage, and facial nerve paralysis [112].

Although myasthenia gravis patients have a higher sensitivity to getting infected by the virus, reports concluded that infection with SARS-Cov-2 can lead to an immune and inflammatory response, which can initiate a myasthenia crisis [112]. It has been reported that COVID-19 patients have a variety of neurological symptoms; however, further research is required to clarify the pathogenic mechanism/s behind each of these muscle disorders

## 5. Involvement of the Olfactory Nerve in SARS-CoV-2 Infection

This section discusses the correlation between COVID-19 and smell dysfunction (another nonspecific symptom) and its underlying mechanisms. Although the exact mechanisms are still unclear, it has been proposed that the involvement of the olfactory bulb or damage to the olfactory cells in the nasal cavity may be affected by COVID-19 [113].

Multiple reasons confirm the fact that the nasal cavity is vulnerable to infection by SARS-CoV-2 [114]. First, viral loads in an infected patient’s nasal cavity were much greater in the pharynx, indicating that the nasal cavity is the major entry site for infection [115]. Secondly, because the SARS-CoV-2 is spread by respiratory droplets, the nasal mucosa (particularly the goblet and ciliated cells) is the most common site of infection [116]. Thirdly, the nasolacrimal duct allows the virus to pass from the tears to the nasal cavity and cause infection [117]. Cathepsins are essential for COVID-19 virus entry via endocytosis [118]. The nasal cavity consists of two types of epithelium: 1) Respiratory epithelium and 2) Olfactory epithelium. The respiratory epithelium serves to humidify the air as the continuation of the respiratory tract [119]. Respiratory mucosa also allows for blocking air dust with muciparous goblet cells, preventing dehydration, and exchanging heat and moisture in the nose cavity. In contrast, the function of the olfactory epithelium is to detect odors via the olfactory sensory neurons and their receptors that are placed on their dendritic cilia [120]. It is worth mentioning that many cell types may be found in the olfactory epithelium. The olfactory receptor neuron is the most crucial of them. It is a bipolar cell that, at its basal surface, gives rise to a thin, unmyelinated axon that carries olfactory information to the brain’s center. Many microvilli, known as olfactory cilia, spread into a thick coating of mucus from a single operation that grows outward from the apical surface of the receptor neuron. Normal individual odors are recognized by olfactory receptor neurons (ORN), which are bipolar neurons in the nasal epithelium with a thin dendritic and 10–20 cilia at the end [121]. At the other end of these neurons, numerous unmyelinated axons penetrate the sub-mucosal lamina to link to the olfactory bulb. 

**Figure 2:**
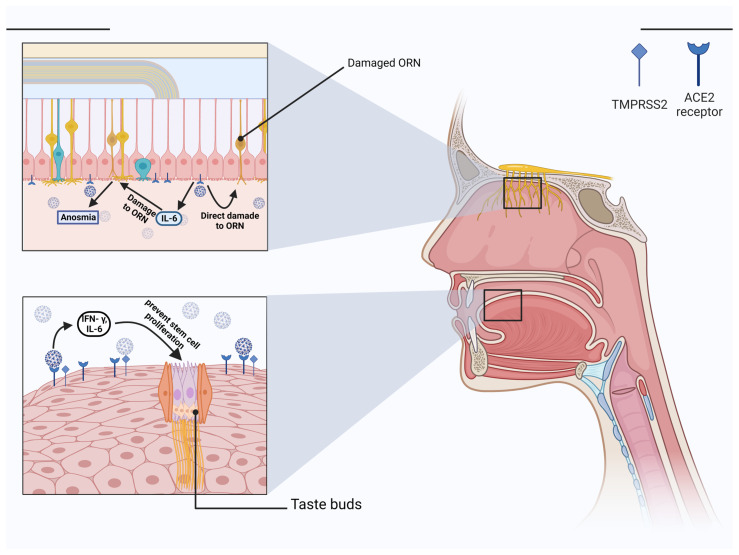
Taste and olfactory dysfunction in COVID-19. IFN- γ and IL-6 are two examples of proinflammatory cytokines that can inhibit stem cells growth and reduce the lifespan of taste bud cells. Another sense that plays a role besides taste is smell. In contrast to ORN and olfactory bulb cells, sustentacle cells express high levels of ACE-2 and TMPRSS2. Loss of cilia on ORN due to infection of the sustentacular cells can interfere with the transmission of the odorous stimulus. Anosmia caused by COVID-19 was also linked to IL-6, proving the importance of this cytokine in the disease’s etiology. (Created with BioRender.com).

Additionally, non-neuronal cells, such as sustentacular cells and Bowman’s glandular cells, are involved in mucus secretion [122]. Which owns different functions but most important, olfactory mucus coats the sensory neurons that sense odorants [123]. RNA sequencing reveals that sustentacular cells, Bowman’s glandular cells, and a limited number of stem cells express ACE-2 and TMPRSS2 significantly, unlike ORN and olfactory bulb cells [122,124]. It is known that the main target of the SARS-CoV-2 is non-neuronal cells, and the reason is that the mean recovery time for smell dysfunction is around two weeks. This period is not compatible with the recovery time of the neuronal cells [124]. The perception of the odors is affected by the damage to the sustentacular and Bowman’s glandular cells via impairing some of the olfactory receptor neuron functions. Damage to the Bowman’s glandular cells may impair mucus production, which is necessary for the elimination of odorous particles, and damage to the sustentacular cells can impede the elimination of volatile compounds. This can interrupt the additional supply for the cilia of the ORN and electrolyte imbalance [122]. This is why damage to the sustentacular cells affects odor perception. In addition, infection of the sustentacular cells can cause ORN to lose their cilia, impairing the transmission of the odorous stimulus [125]. Chemo attractive proteins from IFN-γ, IL-6, and interferon-inducible protein 10 (IP-10) are proinflammatory cytokines that are increased due to entry of a pathogen and recruit monocytes and T-lymphocytes. This inflammatory response that leads to cell death is called pyroptosis [124,126]. Increasing in cytokines and acute-phase reactants can stimulate olfactory receptor neuron death [124,127]. The role of IL-6 in the pathophysiology of COVID-19-induced anosmia was discussed in a study by Netland and Collegues which pointed to encouragement in cytokines, especially IL-6, as a proinflammatory response in the brains of infected mice that changes neuronal signaling to cause anosmia (Figure 2) [128]. Another cause for smell dysfunction is olfactory bulb inflammation, caused by the SARS-CoV-2 using olfactory nerve afferents to reach the olfactory bulb [124]. Additionally, viruses can infect vascular pericytes that are expressing ACE-2, and this can cause inflammation and hypoperfusion [129]. SARS-CoV-2 causes the symptoms of anosmia and hyposmia by interacting with ACE-2 receptors in the nasal cavity with the help of TMPRSS2 and other proteases; the virus’s main targets are the cells that provide it with nutrients and oxygen.

## 6. Ophthalmic Manifestation in COVID-19 

The SARS-CoV-2 has multiple non-specific signs and symptoms. One of its non-specific symptoms is ocular involvement. Many studies have been conducted to investigate whether SARS-CoV-2 has ocular involvement or not. ACE-2 and its aid for entry, TMPRSS2, are the main entry factors for this virus that are expressed in many human organs [110,116]. However, whether cells on the ocular surface express these crucial components for cellular vulnerability to viral infection is still unclear. Western blot analysis has recently revealed that the corneal epithelium expresses TMPRSS2 and ACE-2 during photorefractive keratectomy (PRK) [130]. They also indicated the expression of ACE-2 localization on the corneal epithelium in the most superficial layer, endothelium, and corneal limbus through immunohistochemical staining [131]. 

In contrast, the ACE-2 staining was negative on the corneal stroma. In some cases, the expression of ACE-2 was much higher in the limbus than in the superficial layer of the cornea. They also showed ACE-2 expression on the conjunctival epithelium, but the staining was negative for goblet cells in the epithelium and vascular endothelium [132]. This study proposed that the ocular surface is vulnerable to the SARS-CoV-2; however, conjunctivitis is rare in COVID-19 patients [133]. Lange and Collegues demonstrated that TMPRSS2, ACE-2, and other mediators were not considerably expressed in either healthy or diseased human conjunctival samples, making it unlikely that the SARS-CoV-2 could infect the conjunctiva through these routes [134]. Wu and Collegues conducted a study of 11 patients with ocular manifestations and positive nasopharyngeal RT-PCR and concluded that only 2 patients had positive PCR in the conjunctival swabs. This study proposed that even in patients with ocular manifestations, the positivity of conjunctival swabs is low [135]. Some hypotheses have been proposed as to how the SARS-CoV-2 gets into the ocular secretions; these include direct inoculation of the ocular surface from viral particles, emigration of the virus through the nasolacrimal duct from the pharynx, and hematogenous spread via the lacrimal gland, among others [136]. To clarify this issue, some studies have been conducted. For example, Azzolini and Collegues found SARS-CoV-2 RNA on the ocular surface even when the nasopharyngeal swab was negative [137]. Wang and Collegues demonstrated in a separate investigation that CD147 is a transmembrane glycoprotein that promotes the binding of SARS-CoV-2 to ACE-2. It facilitates the ability for the SARS-CoV-2 to enter the eye and conjunctiva [138]. It has been proposed that the retina has ACE-2 receptors. Twelve patients with positive nasopharyngeal RT-PCR for SARS-CoV-2 underwent optical coherence tomography to detect retinal alterations, and the researchers found hyper-reflective lesions at the level of the ganglion cells and the inner plexiform layer, but no vision loss. Researchers found evidence that SARS-CoV-2 might infect tissues deep inside the eye as well as those on its surface [139]. In the end, it is essential for healthcare workers to put on eye protection during contact with COVID-19 patients, as existing studies are controversial.

## 7. Taste Dysfunction in COVID-19

Taste dysfunction is another example of a non-specific symptom. First, the oral cavity is a crucial entry site for SARS-CoV-2 because multiple entry factors, including ACE-2, TMPRSS2 and furin, are expressed by the oral epithelial cells, taste buds, and salivary glands [140,141]. ACE-2 receptors are expressed all over the oral cavity [110]. Secondly, it has been revealed that the COVID-19 virus may attach to sialic acid receptors, an important component of the saliva, and protect glycoproteins that transport gustatory chemicals in the taste pores from early enzymatic breakdown [142]. Reduced sialylation may affect mucin function by increasing the gustatory threshold and viruses can occupy sialic acid’s binding sites, hence, accelerating the degradation of gustatory particles. Due to the strong association between smell and taste, it is also possible to have problems with both senses simultaneously [143]. However, it was reported that the frequencies of taste dysfunction are higher than olfactory dysfunction [144]. Taste bud cells are columnar sensory cells found in the tongue’s stratified epithelium and have an average life of ten days. They revive continuously from stem cells in the oral cavity [145]. Taste bud cells have receptors that can convert chemical energy into electrical energy [146]. Cranial nerves, such as the facial, glossopharyngeal, and vagus, transfer taste signals from the oral cavity to the brain stem [147]. Damage to any of these cranial nerves can cause gustatory dysfunction. The possible nerve whose damage causes dysgeusia is the chorda tympani [148]. In addition to the mechanisms discussed above, proinflammatory cytokines such as IFN- γ, and IL-6 can prevent stem cell proliferation and shorten the lifetime of taste bud cells (Figure 2) [149]. Another possibility is that the taste bud cells express ACE-2, and this leads to direct infection and cell death [150]. This results from the interaction between the virus and TLR, which induces the apoptotic process in taste bud cells [151]. Yet another possible mechanism is zinc homeostasis impairment of gustatory cells following SARS-CoV-2 infection, which can cause dysgeusia [152]. With all these COVID-19-induced taste dysfunction mechanisms described above, the exact pathophysiology of this disorder remains unknown.

## 8. COVID-19 and the Endocrine System

COVID-19 could exert adverse impacts on different organs, including endocrine dysfunction. The expression of the ACE-2 receptor is responsible for the cellular entrance of COVID-19 via interaction with the S protein. The TMPRSS 2 facilitates the binding of S protein and ACE-2 receptor. ACE-2 receptor expresses widely in the endocrine glands such as the thyroid, pancreas, adrenal glands, pituitary, and testis. Different mechanisms contribute to endocrine damage caused by COVID-19, which include direct viral injury, immune-mediated damage due to cytokine storm, endothelial injury, and the renin–angiotensin–aldosterone system (RAAS) dysfunction. ACE-2 negatively affect RAAS regulation by inactivation of angiotensin (Ang) 1 and 2. The efficacy of ACE-2 reduces during COVID-19 which leads to an elevated level of Ang2 and aldosterone. This RAAS over-activation induces insulin resistance and vascular damage [152,153]. 

In this step, the pathophysiological mechanisms of COVID-19 on the endocrine system are reviewed:

### 8.1. Hypothalamic-Pituitary Axis (HPA)

COVID-19 could damage HPA via direct or indirect injury. The expression of the ACE-2 receptor results in COVID-19 invasion into neurons and glial cells. A virus genome was detected in the autopsy samples which showed edema and neuronal degeneration of the hypothalamus [153,154]. Cytokine storms, including IL-11, IL-6, and TNF-α, cause indirect injury to the HPA. These cytokines increase the secretion of corticotroph-releasing hormone (CRH) and adrenocorticotropic hormone (ACTH) from the hypothalamus and pituitary, respectively, and affect the adrenal to release more cortisol. This neuroinflammation induces hemorrhage, necrosis, and edema in the hypothalamus and pituitary [155,156]. 

Moreover, it has been shown that the spike protein of COVID-19 binds to gonadotropin-releasing hormone receptor (GnRHR) and G-protein-coupled-receptor (GPCR) in the brain and blocks them. GPCRs and GnRHRs act by binding to G proteins. Activation of GPCRs and GNRHRs are responsible for cortisol levels and the release of luteinizing hormone (LH) and follicle-stimulating hormone (FSH), respectively. Inhibition of these receptors disrupts the neuroendocrine system and its effects on peripheral endocrine glands [157]. Furthermore, virus infiltration and cytokine effects impair the hormonal axis mentioned below.

### 8.2. Thyroid 

The thyroid gland regulates metabolism by secreting triiodothyronine (T3) and thyroxine (T4). Studies showed that COVID-19 infects the thyroid and presents in different clinical manifestations such as sick euthyroid syndrome, Graves’ disease, Hashimoto’s syndrome, subacute thyroiditis, and silent thyroiditis. In addition to lymphocyte infiltration in thyroid tissue, ACE-2 receptor β expression provides a target for COVID-19 invasion [152]. Different mechanisms are suggested for thyroid changes in COVID-19 infection. Direct destruction of thyroid follicular and parafollicular cells could induce primary hypothyroidism with a high level of thyroid-stimulating hormone (TSH) and decrease free T4. However, there is no evidence of morphological changes in follicular cells.

On the other hand, the effects of COVID-19 on HPA imply a reduction in TSH level, which leads to a low level of total 3,5,3′-triiodothyronine (TT3) and low free T4. The severity of the infection correlates with the magnitude of the decline. Also, non-thyroidal illness syndrome (NTIS) in critical patients caused low free T3 with normal or low TSH. It is related to deiodinase activity changes due to systemic inflammation [158,159]. 

### 8.3. Pancreas 

ACE-2 and TMPRSS2 are expressed in the pancreas, and its endocrine section can be a target for COVID-19. Studies demonstrated that injury of islet cells leads to diabetes and hyperglycemia. Several mechanisms have been proposed for blood glucose imbalance, including β cells cytotoxicity by COVID-19, systemic inflammation, and glucocorticoid drugs [152,153]. Production of inflammatory factors involving MCP-1, inducible protein-10, and interleukin-1β destruct β cells during COVID-19 infection [153,155].

More factors in β cells facilitate COVID-19 entry. NRP1 and high mobility group box 1 (HMGB1) help bind ACE-2 and COVID-19. HMGB1 is a chromatin regulator that controls ACE-2 expression. Moreover, other molecules similar to SMARCA4, a non-fermenting chromatin-remodeling complex, and RIPK3 as a necroptosis kinase interfere in COVID-19 entry via ACE-2 expression. It has been shown DPP-4, an amino peptide in glucose metabolism, is an alternative receptor for COVID-19 in the pancreas. Due to viral injury, hypoinsulinemia and hyperglycemia can be observed in COVID-19 patients [159]. Shaharuddin and Collegues investigated the expression of inflammatory genes in induced pluripotent stem cells (iPSC)-derived pancreatic cultures infected by COVID-19. Data showed inflammatory genes such as nuclear factor kappa B (NFKB)-1, C-X-C motif chemokine Ligand (CXCL)-12, and signal transducer and activator of transcription (STAT)-3 were upregulated in the infected cells. Additionally, infected endocrine cells of the pancreas showed dysfunction in insulin secretion [160]. 

### 8.4. Adrenal Gland

The expression of ACE-2 receptor and TMPRSS2 zona fasciculate and zona reticularis provides a target for COVID-19 invasion. Besides direct injury of the virus, CD3+ and CD8+ lymphocyte infiltration, acute fibrinoid necrosis in arterioles, and adrenal vein thrombosis damage the adrenal gland. SARS-COV produces molecules similar to ACTH, resulting in presenting auto-antibodies against host ACTH. These antibodies could destroy ACTH and cause adrenal insufficiency (AI). Also, different studies reported clinical manifestations of AI in COVID-19 patients [152,158]. 

## 9. Gastrointestinal (GI) Disorders of COVID19

One of the extrapulmonary manifestations of COVID-19 is GI symptoms. GI symptoms can be presented during different phases of the disease. Most clinical presentations of the disease are diarrhea, loss of appetite, nausea, vomiting, and abdominal pain. Stool examination of patients with GI symptoms demonstrated viral RNA. An autopsy from a patient with COVID-19 has shown necrosis and shedding of GI epithelium from the esophagus to the intestine. Moreover, stenosis of the small intestine has been reported [161]. In addition to GI organs, COVID-19 could lead to liver injury that presents by dysregulation of liver function tests, including aspartate transaminase (AST), alanine transaminase (ALT), gamma-glutamyl transferase (GGT), alkaline phosphatase (ALP), and elevation of bilirubin [162,163]. Different mechanisms are proposed for GI injury:

### 9.1. Direct Injury

ACE-2 and TMPRSS2 are presented on epithelial cells especially on type 2 epithelial cells in the duodenum, jejunum, ileum, cecum, and colon. ACE-2 on enterocytes preserves amino acid homeostasis and antimicrobial molecules. ACE-2 maintains the B0AT1, an amino acid transporter, and controls the uptake of tryptophan in the gut which interferes with immunity, including suppressing inflammatory cytokines, keeping the intestinal junction, and preventing dysbiosis. COVID-19 invasion of enterocytes leads to ACE-2 downregulation which causes RAAS inhibition, a decrease of the anti-inflammatory role of the intestinal mucosa, cellular metabolic condition, and cellular viability. The damage of enterocytes expressing ACE-2 and malabsorption may explain diarrhea in patients with COVID-19 [164]. Yamada and colleagues designed a model of iPSC-derived small intestinal epithelial cells (iPSC-SIECs) infected by COVID-19 and investigated the expression of pro-inflammatory genes electrical resistance (TEER). They demonstrated COVID-19 infection triggers the expression of various inflammatory genes, including CC motif chemokine ligand 2 (CCL2), CCL3, CCL5, CXCL10, IL-6, and IL-1β. Also, it has been reported that TEER was reduced, indicating disruption of tight junction between cells via downregulation of the expression of CLDN1 and ZO-3 genes. The impairment of the intestinal barrier leads to GI symptoms [165]. 

### 9.2. Enteric Nervous System (ENS) Dysfunction

Studies suggested the involvement of ENS in COVID-19 infection. This virus can enter the CNS via ENS with access to splanchnic and vagal nerves. ACE-2 and TMPRSS2 are expressed on the ENS in submucosal and myenteric plexus that can function as a target for COVID-19 invasion and GI damage. Moreover, the inflammation affects ENS, which induces GI symptoms such as nausea and vomiting [166]. 

### 9.3. Immune-Mediated Injury

In some patients with COVID-19 that presented digestive symptoms, the RNA of virus has not been detected. It may suggest the indirect injury of the GI system by COVID-19. Respiratory tract infection by COVID-19 causes intestinal injury through the gut-lung axis. CD4+ T cells derived lung entry small intestine by CC chemokine receptor 9 (CCR9+). CCL25 supports the recruitment of CD4+ T cells. Gut-lung axis impacts the intestinal immunity and disturbs the homeostasis of flora [164,167]. In addition, cytokine storms including IFN-γ, IL-2, IL-7, and ΤΝF-α, cause intestinal inflammation and diarrhea. The detection of fecal calprotectin indicates systemic inflammation in COVID-19, which involves the digestive system [166]. 

### 9.4. Liver Injury 

The elevation of liver enzymes is reported in 7.6%–39% of patients with COVID-19 [168]. Histopathological study showed necrosis and degeneration of hepatic cells during COVID-19 infection [169]. The expression of ACE-2 and TMPRSS2 on hepatocytes, cholangiocytes, and sinusoidal epithelial cells provides a potential for direct injury by COVID-19. This viral invasion disturbs the transportation of bile acid and cholangiocytes barrier.

On the other hand, hypoxia due to pneumonia damages liver function. The increase of inflammatory cytokines such as ILs, IFN-γ, and ΤΝF-α involves in liver damage [162,163]. Table 1 summarizes different mechanisms involved in COVID-19 infection.

## 10. Conclusions

More than three years after the beginning of the pandemic, some of the defining characteristics of SARS-CoV-2 have been identified. The structural components responsible for the pathogenicity of SARS-CoV-2 include S glycoprotein, E glycoprotein, and M and N proteins. During infection, COVID-19 targets two receptors, ACE-2 and TMPRSS2. Viruses may enter cells through several different entry points. Every major body organ and system is vulnerable to the destructive effects of the virus. Anosmia is a characteristic of the virus, and its early onset may indicate a neuro-invasion via the olfactory bulb. The action of COVID-19 on certain organs, such as olfactory cells and taste buds, requires the presence of other molecules, such as cathepsins and sialic acid receptors. 

COVID’s action is not directly on the olfactory nerve but it is known to increase the number of cytokines, which may consequently induce nerve damage. The researchers also discovered that CD147 strengthens the corneal binding between SARS-CoV-2 and ACE-2. Additionally, damage to endothelial cells may also increase nitric oxide (NO), which can raise the risk of thrombosis and lead to cerebrovascular diseases. Similarly, there is little evidence that the incidence of neurodegeneration is rapidly increasing in patients with coronavirus. The effects of COVID-19 on the nervous system and neurological consequences after effective therapy are poorly understood.

**Figure 3:**
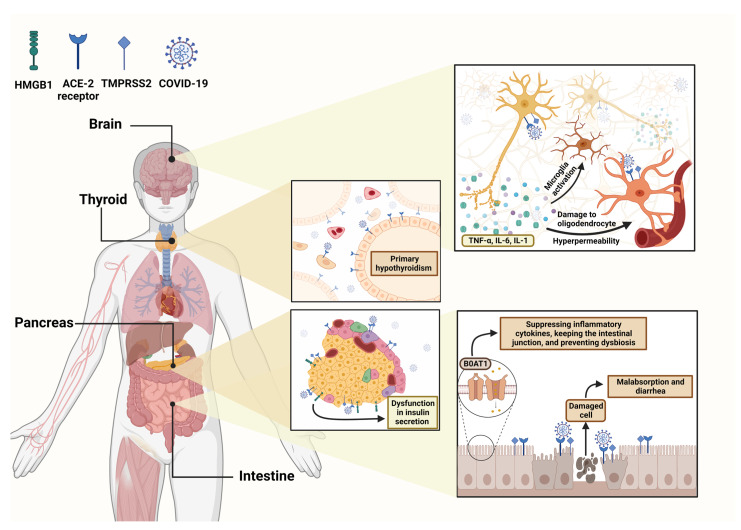
Overview of proposed mechanisms of COVID-19 on damage organ. Besides the respiratory system, the virus may infect and damage a wide variety of other tissues and organs. Virus infection increased the expression levels of IL-6, IL-1, and TNF-α. Even without viral invasion or dissemination, cytokine-driven damage can undermine the BBB. Neurotoxic cytokines can also activate microglia. B0AT1 modulates the absorption of tryptophan in the gut, interfering with immunity by suppressing inflammatory cytokines, maintaining the intestinal junction, and preventing dysbiosis. Also, COVID-19 can infect a follicular cell in thyroid gland and β cells in the pancreas and lead to hypothyroidism and hypoinsulinemia, respectively. (Created with BioRender.com).

In addition, several endocrine glands, including the thyroid, pancreas, adrenal glands, pituitary, and testes, express the ACE-2 receptor. There are a wide range of disease representations for gastrointestinal symptoms. While several clinical studies have detailed the effects of COVID-19 on various organs, elucidating its specific pathophysiological pathways remains challenging. Figure 3 summarizes the proposed mechanisms of infection induced by COVID-19.

## Figures and Tables

**Table 1:** Description of different mechanisms involved in COVID-19 infection.

Systems (Disorders)	Factors Involved	Mechanisms	Reference
CNS (Cerebrovascular diseases)	MCP-1, chemokine, IL-6, IL-1β, TNF-α, and NETs	- The BBB becomes more permeable when MCP-1 levels rise - Contribute to apoptosis in uninfected cells as well as the infiltration and activation of microglia act as a mediator of injury to CNS cells, or even prevent injury altogether - Increase the formation of thrombi	[61,68,80]
Skeletal muscle and Neuromuscular Junction (Myasthenia gravis)	Proinflammatory cytokines	- Can cause constructability enhancement of corrosive molecules, which gives rise to myocyte damage	[109]
Olfactory Nerve (smell dysfunction)	IL-6, IFN-γ, MCP-1, and IP-10	- Impel the expression of multiple acute-phase reactants like CRP, serum amyloid A, haptoglobin, and fibrinogen and can stimulate olfactory receptor neuron death	[160]
Ophthalmic manifestation	ACE-2 receptors, TMPRSS2, CD147,	- Direct inoculation of ocular surface from viral particles, emigration of the virus through nasolacrimal duct from the pharynx, and hematogenous spread via the lacrimal gland	[170]
Taste manifestation	IFN- γ, and IL-6	- Virus binding to the sialic acid receptors that are essential component of the saliva and protect glycoproteins that transfer gustatory molecules in the taste pores from early enzymatic degradation - Can prevent stem cell proliferation and shorten the lifetime of taste bud cells - Zinc homeostasis impairment of gustatory cells	[142,148,151]
Endocrine System (sick euthyroid syndrome, hypothyroidism, diabetes and hyperglycemia)	IL-1, IL-6, and TNFα, the spike protein, MCP-1, inducible protein-10, and inter-leukin-1β	- Leads to an elevated level of Ang2 and aldosterone. This RAAS over-activation induces insulin resistance and vascular damage - Increase the secretion of Corticotroph Releasing Hormone (CRH) and Adrenocorticotropic Hormone (ACTH) from the hypothalamus and pituitary and affect the adrenal to release more cortisol - Binds to gonadotropin-releasing hormone receptor (GnRHR) and G-Protein-Coupled-Receptor (GPCR) in the brain and blocks them - Blood glucose imbalance including β cells cytotoxicity - Inflammatory genes such as Nuclear Factor Kappa B (NFKB)-1, C-X-C motif chemokine Ligand (CXCL)-12, and Signal Transducer and Activator of Transcription (STAT)-3 were upregulated in infected cells	[152,153,155,156,157,160]
GI System (diarrhea, loss of appetite, nausea, vomiting, and abdominal pain)	CCL2, CCL3, CCL5, CXCL10, IL-6, and IL-1β, IFN-γ, IL-2, IL-7, and ΤΝF-α	- Leads to liver injury that presents by dysregulation of liver function tests including Aspartate Transaminase (AST) and Alanine Transaminase (ALT), Gamma-Glutamyl Transferase (GGT), Alkaline phosphatase (ALP), and elevation of bilirubin - Cause intestinal inflammation and diarrhea	[162,163,166,171]

## Data Availability

No new data were created or analyzed in this study. Data sharing is not applicable to this article.

## Abbreviations

Coronavirus Disease 2019	COVID-19
Angiotensin Converting Enzyme-2	ACE-2
Cellular Transmembrane Serine Protease 2	TMPRSS2
Severe acute respiratory syndrome coronavirus 2	SARS-CoV-2
Open Reading Frames	ORF
Nonstructural proteins	nsp
Middle East respiratory syndrome coronavirus	MERS-CoV
Acute Respiratory Distress Syndrome	ARDS
Pattern Recognition Receptors	PRRs
Pathogen-Associated Molecular Patterns	PAMP
Damage-Associated Molecular Patterns	DAMP
Toll-Like Receptors	TLR
RIG-I-Like Receptors	RLR
Scavenger Receptors	SR
Receptor-Binding Domain	RBD
Cluster of Differentiation	CD
C-type lectin CD209L	L-SIGN
Similar protein CD209	DC-SIGN
NOD Like-Receptor Protein	NLRP3
Interleukin	IL
High Density Lipoprotein	HDL
NOD like Receptor	NLR
NOD-Like Receptor Protein	NLRP
Human Coronaviruses	HCoV
Central Nervous System	CNS
Reverse Transcription-Polymerase Chain Reaction	RT-PCR
Tumor Necrosis Factor	TNF
Neutrophil-to-Lymphocyte Ratio	NLR
C-reactive Protein	CRP
Neutrophil Extracellular Traps	NETs
Nitric Oxide Synthase	NOS
Intensive Care Units	ICUs
Cerebro-Spinal Fluid	CSF
Herpes Simplex Virus	HSV
Electroencephalography	EEG
Magnetic Resonance Imaging	MRI
Cytomegalovirus	CMV
acute neurological event	ANE
Guillain–Barré Syndrome	GBS
Multiple Sclerosis	MS
Alzheimer’s disease	AD
Parkinson’s disease	PD
Peripheral Nervous System	PNS
Olfactory Receptor Neurons	ORN
Interferon	IFN
Monocyte Chemoattractant Protein	MCP
Interferon-inducible Protein	IP
Photorefractive Keratectomy	PRK
Renin–Angiotensin–Aldosterone System	RAAS
Hypothalamic-Pituitary Axis	HPA
Corticotroph Releasing Hormone	CRH
Adrenocorticotropic Hormone	ACTH
G-Protein-Coupled-Receptor	GPCR
Luteinizing Hormone	LH
Follicle Stimulating Hormone	FSH
Thyroid Stimulating Hormone	TSH
NonThyroidal Illness Syndrome	NTIS
High mobility group box 1	HMGB1
Nuclear Factor Kappa B	NFKB
C-X-C motif chemokine Ligand	CXCL
Signal Transducer and Activator of Transcription	STAT
Adrenal insufficiency	AI
Gastrointestinal	GI
Aspartate Transaminase	AST
Alanine Transaminase	ALT
Gamma-Glutamyl Transferase	GGT
Alkaline phosphatase	ALP
Small Intestinal Epithelial Cells	iPSC-SIECs
Enteric Nervous System	ENS
CC Chemokine Receptor 9	CCR9+
CC motif Chemokine Ligand	CCL

## References

[1] Ozma M.A., Maroufi P., Khodadadi E., Köse Ş., Esposito I., Ganbarov K., Dao S., Esposito S., Dal T., Zeinalzadeh E. (2020). Clinical manifestation, diagnosis, prevention and control of SARS-CoV-2 (COVID-19) during the outbreak period. Infez. Med..

[2] Hu B., Guo H., Zhou P., Shi Z.L. (2021). Characteristics of SARS-CoV-2 and COVID-19. Nat. Rev. Microbiol..

[3] Lu R., Zhao X., Li J., Niu P., Yang B., Wu H., Wang W., Song H., Huang B., Zhu N. (2020). Genomic characterisation and epidemiology of 2019 novel coronavirus: Implications for virus origins and receptor binding. Lancet.

[4] Hoffmann M., Kleine-Weber H., Schroeder S., Krüger N., Herrler T., Erichsen S., Schiergens T.S., Herrler G., Wu N.-H., Nitsche A. (2020). SARS-CoV-2 cell entry depends on ACE2 and TMPRSS2 and is blocked by a clinically proven protease inhibitor. Cell.

[5] Varatharaj A., Thomas N., Ellul M.A., Davies N.W.S., Pollak T.A., Tenorio E.L., Sultan M., Easton A., Breen G., Zandi M. (2020). Neurological and neuropsychiatric complications of COVID-19 in 153 patients: A UK-wide surveillance study. Lancet Psychiat..

[6] Wang D., Hu B., Hu C., Zhu F., Liu X., Zhang J., Wang B., Xiang H., Cheng Z., Xiong Y. (2020). Clinical characteristics of 138 hospitalized patients with 2019 novel coronavirus–infected pneumonia in Wuhan, China. JAMA.

[7] Ceban F., Ling S., Lui L.M.W., Lee Y., Gill H., Teopiz K.M., Rodrigues N.B., Subramaniapillai M., Di Vincenzo J.D., Cao B. (2022). Fatigue and cognitive impairment in Post-COVID-19 Syndrome: A systematic review and meta-analysis. Brain Behav. Immun..

[8] Ercoli T., Masala C., Pinna I., Orofino G., Solla P., Rocchi L., Defazio G. (2021). Qualitative smell/taste disorders as sequelae of acute COVID-19. Neurol. Sci..

[9] Wee L.E., Chan Y.F.Z., Teo N.W.Y., Cherng B.P.Z., Thien S.Y., Wong H.M., Wijaya L., Toh S.T., Tan T.T. (2020). The role of self-reported olfactory and gustatory dysfunction as a screening criterion for suspected COVID-19. Eur. Arch. Otorhinolaryngol..

[10] Lin L., Jiang X., Zhang Z., Huang S., Zhang Z., Fang Z., Gu Z., Gao L., Shi H., Mai L. (2020). Gastrointestinal symptoms of 95 cases with SARS-CoV-2 infection. Gut.

[11] Puig-Domingo M., Marazuela M., Giustina A. (2020). COVID-19 and endocrine diseases. A statement from the European Society of Endocrinology. Endocrine.

[12] Rehman S.U., Shafique L., Ihsan A., Liu Q. (2020). Evolutionary Trajectory for the Emergence of Novel Coronavirus SARS-CoV-2. Pathogens.

[13] Jiang C., Li X., Ge C., Ding Y., Zhang T., Cao S., Meng L., Lu S. (2021). Molecular detection of SARS-CoV-2 being challenged by virus variation and asymptomatic infection. J. Pharm. Anal..

[14] Chilamakuri R., Agarwal S. (2021). COVID-19: Characteristics and Therapeutics. Cells.

[15] Gorkhali R., Koirala P., Rijal S., Mainali A., Baral A., Bhattarai H.K. (2021). Structure and Function of Major SARS-CoV-2 and SARS-CoV Proteins. Bioinform. Biol. Insights.

[16] Hassan S.S., Attrish D., Ghosh S., Choudhury P.P., Uversky V.N., Aljabali A.A.A., Lundstrom K., Uhal B.D., Rezaei N., Seyran M. (2021). Notable sequence homology of the ORF10 protein introspects the architecture of SARS-CoV-2. Int. J. Biol. Macromol..

[17] Bakhshandeh B., Jahanafrooz Z., Abbasi A., Goli M.B., Sadeghi M., Mottaqi M.S., Zamani M. (2021). Mutations in SARS-CoV-2; Consequences in structure, function, and pathogenicity of the virus. Microb. Pathog..

[18] Camporota L., Chiumello D., Busana M., Gattinoni L., Marini J.J. (2021). Pathophysiology of COVID-19-associated acute respiratory distress syndrome. Lancet Respir. Med..

[19] Okamoto M., Tsukamoto H., Kouwaki T., Seya T., Oshiumi H. (2017). Recognition of Viral RNA by Pattern Recognition Receptors in the Induction of Innate Immunity and Excessive Inflammation During Respiratory Viral Infections. Viral Immunol..

[20] PrabhuDas M.R., Baldwin C.L., Bollyky P.L., Bowdish D.M.E., Drickamer K., Febbraio M., Herz J., Kobzik L., Krieger M., Loike J. (2017). A Consensus Definitive Classification of Scavenger Receptors and Their Roles in Health and Disease. J. Immunol..

[21] Gusev E.Y., Zotova N., Zhuravleva Y.A., Chereshnev V. (2020). Physiological and pathogenic role of scavenger receptors in humans. Med. Immunol..

[22] Wei C., Wan L., Yan Q., Wang X., Zhang J., Yang X., Zhang Y., Fan C., Li D., Deng Y. (2020). HDL-scavenger receptor B type 1 facilitates SARS-CoV-2 entry. Nat. Metab..

[23] Li F. (2016). Structure, Function, and Evolution of Coronavirus Spike Proteins. Annu. Rev. Virol..

[24] Harrison A.G., Lin T., Wang P. (2020). Mechanisms of SARS-CoV-2 Transmission and Pathogenesis. Trends Immunol..

[25] Ji H.L., Zhao R., Matalon S., Matthay M.A. (2020). Elevated Plasmin(ogen) as a Common Risk Factor for COVID-19 Susceptibility. Physiol. Rev..

[26] Evans J.P., Liu S.L. (2021). Role of host factors in SARS-CoV-2 entry. J. Biol. Chem..

[27] Wrapp D., Wang N., Corbett K.S., Goldsmith J.A., Hsieh C.L., Abiona O., Graham B.S., McLellan J.S. (2020). Cryo-EM structure of the 2019-nCoV spike in the prefusion conformation. Science.

[28] Hatmal M.M., Alshaer W., Al-Hatamleh M.A.I., Hatmal M., Smadi O., Taha M.O., Oweida A.J., Boer J.C., Mohamud R., Plebanski M. (2020). Comprehensive Structural and Molecular Comparison of Spike Proteins of SARS-CoV-2, SARS-CoV and MERS-CoV, and Their Interactions with ACE2. Cells.

[29] Shang C., Zhuang X., Zhang H., Li Y., Zhu Y., Lu J., Ge C., Cong J., Li T., Tian M. (2021). Inhibitors of endosomal acidification suppress SARS-CoV-2 replication and relieve viral pneumonia in hACE2 transgenic mice. Virol. J..

[30] Lokhande K.B., Apte G.R., Shrivastava A., Singh A., Pal J.K., Swamy K.V., Gupta R.K. (2022). Sensing the interactions between carbohydrate-binding agents and N-linked glycans of SARS-CoV-2 spike glycoprotein using molecular docking and simulation studies. J. Biomol. Struct. Dyn..

[31] Verma J., Subbarao N. (2021). A comparative study of human betacoronavirus spike proteins: Structure, function and therapeutics. Arch. Virol..

[32] Mycroft-West C.J., Su D., Pagani I., Rudd T.R., Elli S., Gandhi N.S., Guimond S.E., Miller G.J., Meneghetti M.C.Z., Nader H.B. (2020). Heparin Inhibits Cellular Invasion by SARS-CoV-2: Structural Dependence of the Interaction of the Spike S1 Receptor-Binding Domain with Heparin. Thromb. Haemost..

[33] Clausen T.M., Sandoval D.R., Spliid C.B., Pihl J., Perrett H.R., Painter C.D., Narayanan A., Majowicz S.A., Kwong E.M., McVicar R.N. (2020). SARS-CoV-2 Infection Depends on Cellular Heparan Sulfate and ACE2. Cell.

[34] Cagno V., Tseligka E.D., Jones S.T., Tapparel C. (2019). Heparan Sulfate Proteoglycans and Viral Attachment: True Receptors or Adaptation Bias?. Viruses.

[35] Seyran M., Takayama K., Uversky V.N., Lundstrom K., Palù G., Sherchan S.P., Attrish D., Rezaei N., Aljabali A.A.A., Ghosh S. (2021). The structural basis of accelerated host cell entry by SARS-CoV-2. Febs. J..

[36] Dakal T.C. (2021). SARS-CoV-2 attachment to host cells is possibly mediated via RGD-integrin interaction in a calcium-dependent manner and suggests pulmonary EDTA chelation therapy as a novel treatment for COVID 19. Immunobiology.

[37] Hassanpour M., Rezaie J., Nouri M., Panahi Y. (2020). The role of extracellular vesicles in COVID-19 virus infection. Infect. Genet Evol..

[38] Elrashdy F., Aljaddawi A.A., Redwan E.M., Uversky V.N. (2021). On the potential role of exosomes in the COVID-19 reinfection/reactivation opportunity. J. Biomol. Struct. Dyn..

[39] Xia X., Yuan P., Liu Y., Wang Y., Cao W., Zheng J.C. (2021). Emerging roles of extracellular vesicles in COVID-19, a double-edged sword?. Immunology.

[40] Gurunathan S., Kang M.H., Kim J.H. (2021). Diverse Effects of Exosomes on COVID-19: A Perspective of Progress From Transmission to Therapeutic Developments. Front. Immunol..

[41] Fenizia C., Galbiati S., Vanetti C., Vago R., Clerici M., Tacchetti C., Daniele T. (2021). SARS-CoV-2 Entry: At the Crossroads of CD147 and ACE2. Cells.

[42] Krejner-Bienias A., Grzela K., Grzela T. (2021). DPP4 Inhibitors and COVID-19-Holy Grail or Another Dead End?. Arch. Immunol. Ther. Exp..

[43] Kočar E., Režen T., Rozman D. (2021). Cholesterol, lipoproteins, and COVID-19: Basic concepts and clinical applications. Biochim. Biophys. Acta Mol. Cell Biol. Lipids.

[44] Amraei R., Yin W., Napoleon M.A., Suder E.L., Berrigan J., Zhao Q., Olejnik J., Chandler K.B., Xia C., Feldman J. (2021). CD209L/L-SIGN and CD209/DC-SIGN Act as Receptors for SARS-CoV-2. ACS Cent Sci..

[45] Lo M.W., Kemper C., Woodruff T.M. (2020). COVID-19: Complement, Coagulation, and Collateral Damage. J. Immunol..

[46] Perico L., Benigni A., Casiraghi F., Ng L.F.P., Renia L., Remuzzi G. (2021). Immunity, endothelial injury and complement-induced coagulopathy in COVID-19. Nat. Rev. Nephrol..

[47] Aboudounya M.M., Heads R.J. (2021). COVID-19 and Toll-Like Receptor 4 (TLR4): SARS-CoV-2 May Bind and Activate TLR4 to Increase ACE2 Expression, Facilitating Entry and Causing Hyperinflammation. Mediat. Inflamm..

[48] Choudhury A., Mukherjee S. (2020). In silico studies on the comparative characterization of the interactions of SARS-CoV-2 spike glycoprotein with ACE-2 receptor homologs and human TLRs. J. Med. Viro.l.

[49] Moreno-Eutimio M.A., López-Macías C., Pastelin-Palacios R. (2020). Bioinformatic analysis and identification of single-stranded RNA sequences recognized by TLR7/8 in the SARS-CoV-2, SARS-CoV, and MERS-CoV genomes. Microbes Infect..

[50] de Marcken M., Dhaliwal K., Danielsen A.C., Gautron A.S., Dominguez-Villar M. (2019). TLR7 and TLR8 activate distinct pathways in monocytes during RNA virus infection. Sci. Signal..

[51] Onomoto K., Onoguchi K., Yoneyama M. (2021). Regulation of RIG-I-like receptor-mediated signaling: Interaction between host and viral factors. Cell Mol. Immunol..

[52] Rodrigues T.S., de Sá K.S.G., Ishimoto A.Y., Becerra A., Oliveira S., Almeida L., Gonçalves A.V., Perucello D.B., Andrade W.A., Castro R. (2021). Inflammasomes are activated in response to SARS-CoV-2 infection and are associated with COVID-19 severity in patients. J. Exp. Med..

[53] Gusev E.Y., Zotova N.V. (2019). Cellular Stress and General Pathological Processes. Curr. Pharm. Des..

[54] Gusev E., Sarapultsev A., Hu D., Chereshnev V. (2021). Problems of Pathogenesis and Pathogenetic Therapy of COVID-19 from the Perspective of the General Theory of Pathological Systems (General Pathological Processes). Int. J. Mol. Sci..

[55] Papanikolaou V., Chrysovergis A., Ragos V., Tsiambas E., Katsinis S., Manoli A., Papouliakos S., Roukas D., Mastronikolis S., Peschos D. (2022). From delta to Omicron: S1-RBD/S2 mutation/deletion equilibrium in SARS-CoV-2 defined variants. Gene.

[56] Sardu C., Gambardella J., Morelli M.B., Wang X., Marfella R., Santulli G. (2020). Hypertension, Thrombosis, Kidney Failure, and Diabetes: Is COVID-19 an Endothelial Disease? A Comprehensive Evaluation of Clinical and Basic Evidence. J. Clin. Med..

[57] Ding Y., He L., Zhang Q., Huang Z., Che X., Hou J., Wang H., Shen H., Qiu L., Li Z. (2004). Organ distribution of severe acute respiratory syndrome (SARS) associated coronavirus (SARS-CoV) in SARS patients: Implications for pathogenesis and virus transmission pathways. J. Pathol..

[58] Arbour N., Day R., Newcombe J., Talbot P.J. (2000). Neuroinvasion by human respiratory coronaviruses. J. Virol..

[59] Edwards J.A., Denis F., Talbot P.J. (2000). Activation of glial cells by human coronavirus OC43 infection. J. Neuroimmunol..

[60] Stamatovic S.M., Shakui P., Keep R.F., Moore B.B., Kunkel S.L., Van Rooijen N., Andjelkovic A.V. (2005). Monocyte chemoattractant protein-1 regulation of blood-brain barrier permeability. J. Cereb. Blood Flow. Metab..

[61] Glass W.G., Subbarao K., Murphy B., Murphy P.M. (2004). Mechanisms of host defense following severe acute respiratory syndrome-coronavirus (SARS-CoV) pulmonary infection of mice. J. Immunol..

[62] Kandemirli S.G., Dogan L., Sarikaya Z.T., Kara S., Akinci C., Kaya D., Kaya Y., Yildirim D., Tuzuner F., Yildirim M.S. (2020). Brain MRI Findings in Patients in the Intensive Care Unit with COVID-19 Infection. Radiology.

[63] Torabi A., Mohammadbagheri E., Akbari Dilmaghani N., Bayat A.H., Fathi M., Vakili K., Alizadeh R., Rezaeimirghaed O., Hajiesmaeili M., Ramezani M. (2020). Proinflammatory Cytokines in the Olfactory Mucosa Result in COVID-19 Induced Anosmia. ACS Chem. Neurosci..

[64] Solomon I.H., Normandin E., Bhattacharyya S., Mukerji S.S., Keller K., Ali A.S., Adams G., Hornick J.L., Padera R.F., Sabeti P. (2020). Neuropathological Features of COVID-19. N. Engl. J. Med..

[65] Murray R.S., Brown B., Brian D., Cabirac G.F. (1992). Detection of coronavirus RNA and antigen in multiple sclerosis brain. Ann. Neurol..

[66] Netland J., Meyerholz D.K., Moore S., Cassell M., Perlman S. (2008). Severe acute respiratory syndrome coronavirus infection causes neuronal death in the absence of encephalitis in mice transgenic for human ACE2. J. Virol..

[67] Jacomy H., Fragoso G., Almazan G., Mushynski W.E., Talbot P.J. (2006). Human coronavirus OC43 infection induces chronic encephalitis leading to disabilities in BALB/C mice. Virology.

[68] Robertson J., Beaulieu J.M., Doroudchi M.M., Durham H.D., Julien J.P., Mushynski W.E. (2001). Apoptotic death of neurons exhibiting peripherin aggregates is mediated by the proinflammatory cytokine tumor necrosis factor-alpha. J. Cell Biol..

[69] Wan Y., Shang J., Graham R., Baric R.S., Li F. (2020). Receptor Recognition by the Novel Coronavirus from Wuhan: An Analysis Based on Decade-Long Structural Studies of SARS Coronavirus. J. Virol..

[70] Monteil V., Kwon H., Prado P., Hagelkrüys A., Wimmer R.A., Stahl M., Leopoldi A., Garreta E., Hurtado Del Pozo C., Prosper F. (2020). Inhibition of SARS-CoV-2 Infections in Engineered Human Tissues Using Clinical-Grade Soluble Human ACE2. Cell.

[71] Yang X.H., Deng W., Tong Z., Liu Y.X., Zhang L.F., Zhu H., Gao H., Huang L., Liu Y.L., Ma C.M. (2007). Mice transgenic for human angiotensin-converting enzyme 2 provide a model for SARS coronavirus infection. Comp. Med..

[72] Wang S., Le T.Q., Kurihara N., Chida J., Cisse Y., Yano M., Kido H. (2010). Influenza virus-cytokine-protease cycle in the pathogenesis of vascular hyperpermeability in severe influenza. J. Infect. Dis..

[73] Ouattara L.A., Barin F., Barthez M.A., Bonnaud B., Roingeard P., Goudeau A., Castelnau P., Vernet G., Paranhos-Baccalà G., Komurian-Pradel F. (2011). Novel human reovirus isolated from children with acute necrotizing encephalopathy. Emerg. Infect. Dis..

[74] Allan S.M., Rothwell N.J. (2001). Cytokines and acute neurodegeneration. Nat. Rev. Neurosci..

[75] Ahmad I., Rathore F.A. (2020). Neurological manifestations and complications of COVID-19: A literature review. J. Clin. Neurosci..

[76] Andalib S., Biller J., Di Napoli M., Moghimi N., McCullough L.D., Rubinos C.A., O’Hana Nobleza C., Azarpazhooh M.R., Catanese L., Elicer I. (2021). Peripheral Nervous System Manifestations Associated with COVID-19. Curr. Neurol. Neurosci. Rep..

[77] Yaghi S., Ishida K., Torres J., Mac Grory B., Raz E., Humbert K., Henninger N., Trivedi T., Lillemoe K., Alam S. (2020). SARS-CoV-2 and Stroke in a New York Healthcare System. Stroke.

[78] Klok F.A., Kruip M., van der Meer N.J.M., Arbous M.S., Gommers D., Kant K.M., Kaptein F.H.J., van Paassen J., Stals M.A.M., Huisman M.V. (2020). Incidence of thrombotic complications in critically ill ICU patients with COVID-19. Thromb. Res..

[79] Fois A.G., Paliogiannis P., Scano V., Cau S., Babudieri S., Perra R., Ruzzittu G., Zinellu E., Pirina P., Carru C. (2020). The Systemic Inflammation Index on Admission Predicts In-Hospital Mortality in COVID-19 Patients. Molecules.

[80] Middleton E.A., He X.Y., Denorme F., Campbell R.A., Ng D., Salvatore S.P., Mostyka M., Baxter-Stoltzfus A., Borczuk A.C., Loda M. (2020). Neutrophil extracellular traps contribute to immunothrombosis in COVID-19 acute respiratory distress syndrome. Blood.

[81] Akerström S., Gunalan V., Keng C.T., Tan Y.J., Mirazimi A. (2009). Dual effect of nitric oxide on SARS-CoV replication: Viral RNA production and palmitoylation of the S protein are affected. Virology.

[82] Kochi A.N., Tagliari A.P., Forleo G.B., Fassini G.M., Tondo C. (2020). Cardiac and arrhythmic complications in patients with COVID-19. J. Cardiovasc. Electrophysiol..

[83] Sharifian-Dorche M., Huot P., Osherov M., Wen D., Saveriano A., Giacomini P.S., Antel J.P., Mowla A. (2020). Neurological complications of coronavirus infection; a comparative review and lessons learned during the COVID-19 pandemic. J. Neurol. Sci..

[84] Oxley T.J., Mocco J., Majidi S., Kellner C.P., Shoirah H., Singh I.P., De Leacy R.A., Shigematsu T., Ladner T.R., Yaeger K.A. (2020). Large-Vessel Stroke as a Presenting Feature of Covid-19 in the Young. N. Engl. J. Med..

[85] Meyfroidt G., Kurtz P., Sonneville R. (2020). Critical care management of infectious meningitis and encephalitis. Intensive Care Med..

[86] Wu Y., Xu X., Chen Z., Duan J., Hashimoto K., Yang L., Liu C., Yang C. (2020). Nervous system involvement after infection with COVID-19 and other coronaviruses. Brain Behav. Immun..

[87] Benameur K., Agarwal A., Auld S.C., Butters M.P., Webster A.S., Ozturk T., Howell J.C., Bassit L.C., Velasquez A., Schinazi R.F. (2020). Encephalopathy and Encephalitis Associated with Cerebrospinal Fluid Cytokine Alterations and Coronavirus Disease, Atlanta, Georgia, USA, 2020. Emerg. Infect. Dis..

[88] Cag Y., Erdem H., Leib S., Defres S., Kaya S., Larsen L., Poljak M., Ozturk-Engin D., Barsic B., Argemi X. (2016). Managing atypical and typical herpetic central nervous system infections: Results of a multinational study. Clin. Microbiol. Infect..

[89] Hepburn M., Mullaguri N., George P., Hantus S., Punia V., Bhimraj A., Newey C.R. (2021). Acute Symptomatic Seizures in Critically Ill Patients with COVID-19: Is There an Association?. Neurocrit. Care.

[90] Lu L., Xiong W., Liu D., Liu J., Yang D., Li N., Mu J., Guo J., Li W., Wang G. (2020). New onset acute symptomatic seizure and risk factors in coronavirus disease 2019: A retrospective multicenter study. Epilepsia.

[91] Nguyen T.P., Taylor R.S., Renwanz Boyle A.G. (2021). Guillain Barre Syndrome (Nursing).

[92] Rahimi K. (2020). Guillain-Barre syndrome during COVID-19 pandemic: An overview of the reports. Neurol. Sci..

[93] Sancho-Saldaña A., Lambea-Gil Á., Liesa J.L.C., Caballo M.R.B., Garay M.H., Celada D.R., Serrano-Ponz M. (2020). Guillain-Barré syndrome associated with leptomeningeal enhancement following SARS-CoV-2 infection. Clin. Med..

[94] Tatu L., Nono S., Grácio S., Koçer S. (2021). Guillain-Barré syndrome in the COVID-19 era: Another occasional cluster?. J. Neurol..

[95] Wang F., Kream R.M., Stefano G.B. (2020). Long-Term Respiratory and Neurological Sequelae of COVID-19. Med. Sci. Monit..

[96] Lukiw W.J., Pogue A., Hill J.M. (2022). SARS-CoV-2 Infectivity and Neurological Targets in the Brain. Cell Mol. Neurobiol..

[97] Lassmann H. (2018). Multiple Sclerosis Pathology. Cold Spring Harb. Perspect. Med..

[98] Kempuraj D., Selvakumar G.P., Ahmed M.E., Raikwar S.P., Thangavel R., Khan A., Zaheer S.A., Iyer S.S., Burton C., James D. (2020). COVID-19, Mast Cells, Cytokine Storm, Psychological Stress, and Neuroinflammation. Neuroscientist.

[99] Zanin L., Saraceno G., Panciani P.P., Renisi G., Signorini L., Migliorati K., Fontanella M.M. (2020). SARS-CoV-2 can induce brain and spine demyelinating lesions. Acta Neurochir..

[100] Boziki M.K., Mentis A.A., Shumilina M., Makshakov G., Evdoshenko E., Grigoriadis N. (2020). COVID-19 Immunopathology and the Central Nervous System: Implication for Multiple Sclerosis and Other Autoimmune Diseases with Associated Demyelination. Brain Sci..

[101] Sormani M.P. (2020). An Italian programme for COVID-19 infection in multiple sclerosis. Lancet Neurol..

[102] Fuchs V., Kutza M., Wischnewski S., Deigendesch N., Lutz L., Kulsvehagen L., Ricken G., Kappos L., Tzankov A., Hametner S. (2021). Presence of SARS-CoV-2 Transcripts in the Choroid Plexus of MS and Non-MS Patients With COVID-19. Neurol. Neuroimmunol. Neuroinflamm..

[103] Krajcovicova L., Klobusiakova P., Rektorova I. (2019). Gray Matter Changes in Parkinson’s and Alzheimer’s Disease and Relation to Cognition. Curr. Neurol. Neurosci. Rep..

[104] McAlpine L.S., Fesharaki-Zadeh A., Spudich S. (2021). Coronavirus disease 2019 and neurodegenerative disease: What will the future bring?. Curr. Opin. Psychiatry.

[105] Beitz J.M. (2014). Parkinson’s disease: A review. Front. Biosci. (Schol. Ed.).

[106] Bernaus A., Blanco S., Sevilla A. (2020). Glia Crosstalk in Neuroinflammatory Diseases. Front. Cell Neurosci..

[107] Van Bulck M., Sierra-Magro A., Alarcon-Gil J., Perez-Castillo A., Morales-Garcia J.A. (2019). Novel Approaches for the Treatment of Alzheimer’s and Parkinson’s Disease. Int. J. Mol. Sci..

[108] Ponsen M.M., Stoffers D., Booij J., van Eck-Smit B.L., Wolters E., Berendse H.W. (2004). Idiopathic hyposmia as a preclinical sign of Parkinson’s disease. Ann. Neurol..

[109] Welch C., Greig C., Masud T., Wilson D., Jackson T.A. (2020). COVID-19 and Acute Sarcopenia. Aging Dis..

[110] Zhou P., Yang X.-L., Wang X.-G., Hu B., Zhang L., Zhang W., Si H.-R., Zhu Y., Li B., Huang C.-L. (2020). A pneumonia outbreak associated with a new coronavirus of probable bat origin. Nature.

[111] Zhang W., Xu L., Luo T., Wu F., Zhao B., Li X. (2020). The etiology of Bell’s palsy: A review. J. Neurol..

[112] Koc G., Odabasi Z., Tan E. (2020). Myasthenic Syndrome Caused by Hydroxychloroquine Used for COVID-19 Prophylaxis. J. Clin. Neuromuscul Dis.

[113] Agyeman A.A., Chin K.L., Landersdorfer C.B., Liew D., Ofori-Asenso R. (2020). Smell and taste dysfunction in patients with COVID-19: A systematic review and meta-analysis. Mayo Clin. Proc..

[114] Meng X., Deng Y., Dai Z., Meng Z. (2020). COVID-19 and anosmia: A review based on up-to-date knowledge. Am. J. Otolaryngol..

[115] Zou L., Ruan F., Huang M., Liang L., Huang H., Hong Z., Yu J., Kang M., Song Y., Xia J. (2020). SARS-CoV-2 viral load in upper respiratory specimens of infected patients. N. Engl. J. Med..

[116] Sungnak W., Huang N., Bécavin C., Berg M., Queen R., Litvinukova M., Talavera-López C., Maatz H., Reichart D., Sampaziotis F. (2020). SARS-CoV-2 entry factors are highly expressed in nasal epithelial cells together with innate immune genes. Nat. Med..

[117] Colavita F., Lapa D., Carletti F., Lalle E., Bordi L., Marsella P., Nicastri E., Bevilacqua N., Giancola M.L., Corpolongo A. (2020). SARS-CoV-2 isolation from ocular secretions of a patient with COVID-19 in Italy with prolonged viral RNA detection. Ann. Intern. Med..

[118] Ou X., Liu Y., Lei X., Li P., Mi D., Ren L., Guo L., Guo R., Chen T., Hu J. (2020). Characterization of spike glycoprotein of SARS-CoV-2 on virus entry and its immune cross-reactivity with SARS-CoV. Nat. Commun..

[119] Reznik G.K. (1990). Comparative anatomy, physiology, and function of the upper respiratory tract. Environ. Health Perspecives.

[120] Suzuki Y., Schafer J., Farbman A.I. (1995). Phagocytic cells in the rat olfactory epithelium after bulbectomy. Exp. Neurol..

[121] Menini A., Lagostena L., Boccaccio A. (2004). Olfaction: From odorant molecules to the olfactory cortex. News. Physiol. Sci..

[122] Brann D.H., Tsukahara T., Weinreb C., Lipovsek M., Van den Berge K., Gong B., Chance R., Macaulay I.C., Chou H.-J., Fletcher R.B. (2020). Non-neuronal expression of SARS-CoV-2 entry genes in the olfactory system suggests mechanisms underlying COVID-19-associated anosmia. Sci. Adv..

[123] Shirai T., Takase D., Yokoyama J., Nakanishi K., Uehara C., Saito N., Kato-Namba A., Yoshikawa K. (2023). Functions of human olfactory mucus and age-dependent changes. Sci. Rep..

[124] Eshraghi A.A., Mirsaeidi M., Davies C., Telischi F.F., Chaudhari N., Mittal R. (2020). Potential mechanisms for COVID-19 induced anosmia and dysgeusia. Front Physiol..

[125] Bilinska K., Butowt R. (2020). Anosmia in COVID-19: A bumpy road to establishing a cellular mechanism. ACS Chem. Neurosci..

[126] Butowt R., von Bartheld C.S. (2021). Anosmia in COVID-19: Underlying mechanisms and assessment of an olfactory route to brain infection. Neuroscientist.

[127] Cazzolla A.P., Lovero R., Lo Muzio L., Testa N.F., Schirinzi A., Palmieri G., Pozzessere P., Procacci V., Di Comite M., Ciavarella D. (2020). Taste and smell disorders in COVID-19 patients: Role of interleukin-6. ACS Chem. Neurosci..

[128] Mastrangelo A., Bonato M., Cinque P. (2021). Smell and taste disorders in COVID-19: From pathogenesis to clinical features and outcomes. Neurosci. Lett..

[129] Ohkubo K., Lee C.H., Baraniuk J.N., Merida M., Hausfeld J.N., Kaliner M.A. (1994). Angiotensin-converting enzyme in the human nasal mucosa. Am. J. Respir. Cell Mol. Biol..

[130] Li W., Moore M.J., Vasilieva N., Sui J., Wong S.K., Berne M.A., Somasundaran M., Sullivan J.L., Luzuriaga K., Greenough T.C. (2003). Angiotensin-converting enzyme 2 is a functional receptor for the SARS coronavirus. Nature.

[131] He L., Ding Y., Zhang Q., Che X., He Y., Shen H., Wang H., Li Z., Zhao L., Geng J. (2006). Expression of elevated levels of pro-inflammatory cytokines in SARS-CoV-infected ACE2+ cells in SARS patients: Relation to the acute lung injury and pathogenesis of SARS. J. Pathol..

[132] Hamming I., Timens W., Bulthuis M., Lely A., Navis G.V., van Goor H. (2004). Tissue distribution of ACE2 protein, the functional receptor for SARS coronavirus. A first step in understanding SARS pathogenesis. J. Pathol..

[133] Zhou L., Xu Z., Castiglione G.M., Soiberman U.S., Eberhart C.G., Duh E.J. (2020). ACE2 and TMPRSS2 are expressed on the human ocular surface, suggesting susceptibility to SARS-CoV-2 infection. Ocul. Surf..

[134] Lange C., Wolf J., Auw-Haedrich C., Schlecht A., Boneva S., Lapp T., Horres R., Agostini H., Martin G., Reinhard T. (2020). Expression of the COVID-19 receptor ACE2 in the human conjunctiva. J. Med. Virol..

[135] Wu P., Duan F., Luo C., Liu Q., Qu X., Liang L., Wu K. (2020). Characteristics of ocular findings of patients with coronavirus disease 2019 (COVID-19) in Hubei Province, China. JAMA Ophthalmol..

[136] Seah I., Agrawal R. (2020). Can the coronavirus disease 2019 (COVID-19) affect the eyes? A review of coronaviruses and ocular implications in humans and animals. Ocul. Immunol. Inflamm..

[137] Liu Z., Sun C.B. (2020). Conjunctiva is not a preferred gateway of entry for SARS-CoV-2 to infect respiratory tract. J. Med. Virol..

[138] Wang K., Chen W., Zhou Y.-S., Lian J.-Q., Zhang Z., Du P., Gong L., Zhang Y., Cui H.-Y., Geng J.-J. (2020). CD147-spike proteinis a novel route for SARS-CoV-2 infection to host cells. Signal Transduct Target Ther.

[139] Marinho P.M., Marcos A.A., Romano A.C., Nascimento H., Belfort R. (2020). Retinal findings in patients with COVID-19. Lancet.

[140] Usami Y., Hirose K., Okumura M., Toyosawa S., Sakai T. (2021). Brief communication: Immunohistochemical detection of ACE2 in human salivary gland. Oral. Sci. Int..

[141] Lechien J.R., Radulesco T., Calvo-Henriquez C., Chiesa-Estomba C.M., Hans S., Barillari M.R., Cammaroto G., Descamps G., Hsieh J., Vaira L. (2021). ACE2 & TMPRSS2 expressions in head & neck tissues: A systematic review. Head Neck Pathol..

[142] Milanetti E., Miotto M., Di Rienzo L., Nagaraj M., Monti M., Golbek T.W., Gosti G., Roeters S.J., Weidner T., Otzen D.E. (2021). In-silico evidence for a two receptor based strategy of SARS-CoV-2. Front. Mol. Biosci..

[143] Small D.M., Prescott J. (2005). Odor/taste integration and the perception of flavor. Exp. Brain Res..

[144] Roper S.D., Chaudhari N. (2017). Taste buds: Cells, signals and synapses. Nat. Rev. Neurosci..

[145] Roper S.D. (2013). Taste buds as peripheral chemosensory processors. Semin Cell Dev. Biol..

[146] Breslin P.A. (2013). An evolutionary perspective on food and human taste. Curr. Biol..

[147] Finsterer J., Stollberger C. (2020). Causes of hypogeusia/hyposmia in SARS-CoV2 infected patients. J. Med. Virol..

[148] Cohn Z.J., Kim A., Huang L., Brand J., Wang H. (2010). Lipopolysaccharide-induced inflammation attenuates taste progenitor cell proliferation and shortens the life span of taste bud cells. BMC Neurosci..

[149] Xu H., Zhong L., Deng J., Peng J., Dan H., Zeng X. (2020). High expression of ACE2 receptor of 2019-nCoV onthe epitelial cells of oral mucosa. Int. J. Oral. Sci..

[150] Wang H., Zhou M., Brand J., Huang L. (2009). Inflammation and taste disorders: Mechanisms in taste buds. Ann. N. Y Acad. Sci..

[151] Clark H.L., Jhingran A., Sun Y., Vareechon C., de Jesus Carrion S., Skaar E.P., Chazin W.J., Calera J.A., Hohl T.M., Pearlman E. (2016). Zinc and manganese chelation by neutrophil S100A8/A9 (calprotectin) limits extracellular Aspergillus fumigatus hyphal growth and corneal infection. J. Immunol..

[152] Esmaeilzadeh A., Elahi R., Siahmansouri A., Maleki A.J., Moradi A. (2022). Endocrine and metabolic complications of COVID-19: Lessons learned and future prospects. J. Mol. Endocrinol..

[153] Abdel-Moneim A., Hosni A. (2021). Insights into the possible impact of COVID-19 on the endocrine system. Arch. Physiol. Biochem..

[154] Lechien J.R., Chiesa-Estomba C.M., De Siati D.R., Horoi M., Le Bon S.D., Rodriguez A., Dequanter D., Blecic S., El Afia F., Distinguin L. (2020). Olfactory and gustatory dysfunctions as a clinical presentation of mild-to-moderate forms of the coronavirus disease (COVID-19): A multicenter European study. Eur. Arch. Otorhinolaryngol..

[155] Garg M.K., Gopalakrishnan M., Yadav P., Misra S. (2020). Endocrine Involvement in COVID-19: Mechanisms, Clinical Features, and Implications for Care. Indian J. Endocrinol. Metab..

[156] Hornick M.G., Olson M.E., Jadhav A.L. (2022). SARS-CoV-2 Psychiatric Sequelae: A Review of Neuroendocrine Mechanisms and Therapeutic Strategies. Int. J. Neuropsychopharmacol..

[157] Elkazzaz M., Ahmed A., Abo-Amer Y.E., Hydara T., Haikal A., Razek D., Eltayb W.A., Wang X., Karpiński T.M., Hamza D. (2022). In Silico Discovery of GPCRs and GnRHRs as Novel Binding Receptors of SARS-CoV-2 Spike Protein Could Explain Neuroendocrine Disorders in COVID-19. Vaccines.

[158] Piticchio T., Le Moli R., Tumino D., Frasca F. (2021). Relationship between betacoronaviruses and the endocrine system: A new key to understand the COVID-19 pandemic-A comprehensive review. J. Endocrinol. Investig..

[159] Chen W., Tian Y., Li Z., Zhu J., Wei T., Lei J. (2021). Potential Interaction Between SARS-CoV-2 and Thyroid: A Review. Endocrinology.

[160] Shaharuddin S.H., Wang V., Santos R.S., Gross A., Wang Y., Jawanda H., Zhang Y., Hasan W., Garcia G., Arumugaswami V. (2021). Deleterious Effects of SARS-CoV-2 Infection on Human Pancreatic Cells. Front. Cell Infect. Microbiol..

[161] Saeed U., Piracha Z.Z., Uppal S.R., Waheed Y., Uppal R. (2022). SARS-CoV-2 induced hepatic injuries and liver complications. Front. Cell Infect. Microbiol..

[162] Campbell P.T., Fix O.K. (2023). Coronavirus Disease-2019 and Implications on the Liver. Clin. Liver Dis..

[163] Alnamshan M.M. (2022). Potential histopathological and immunological effects of SARS-CoV-2 on the liver. Braz. J. Biol..

[164] Li S., Zhou Y., Yan D., Wan Y. (2022). An Update on the Mutual Impact between SARS-CoV-2 Infection and Gut Microbiota. Viruses.

[165] Yamada S., Noda T., Okabe K., Yanagida S., Nishida M., Kanda Y. (2022). SARS-CoV-2 induces barrier damage and inflammatory responses in the human iPSC-derived intestinal epithelium. J. Pharmacol. Sci..

[166] Marasco G., Lenti M.V., Cremon C., Barbaro M.R., Stanghellini V., Di Sabatino A., Barbara G. (2021). Implications of SARS-CoV-2 infection for neurogastroenterology. Neurogastroenterol. Motil..

[167] Chen T.H., Hsu M.T., Lee M.Y., Chou C.K. (2022). Gastrointestinal Involvement in SARS-CoV-2 Infection. Viruses.

[168] Zhong P., Xu J., Yang D., Shen Y., Wang L., Feng Y., Du C., Song Y., Wu C., Hu X. (2020). COVID-19-associated gastrointestinal and liver injury: Clinical features and potential mechanisms. Signal. Transduct. Target Ther..

[169] Dawood R.M., Maher Salum G., Abd El-Meguid M. (2022). The Impact of COVID-19 on Liver Injury. Am J Med Sci..

[170] Willcox M.D., Walsh K., Nichols J.J., Morgan P.B., Jones L.W. (2020). The ocular surface, coronaviruses and COVID-19. Clin. Exp. Optom..

[171] Lei H.Y., Ding Y.H., Nie K., Dong Y.M., Xu J.H., Yang M.L., Liu M.Q., Wei L., Nasser M.I., Xu L.Y. (2021). Potential effects of SARS-CoV-2 on the gastrointestinal tract and liver. Biomed. Pharm..

